# Chronic Pain Secondary to a Cervical Intramedullary Ependymoma: A Case Report

**DOI:** 10.7759/cureus.20277

**Published:** 2021-12-08

**Authors:** Ali Atoot, Christine Ha, Motasem Abul-Huda, Fawzi Kaawar, Adam Atoot, Surjya Sen, Gary Panagiotakis, Mark Schlesinger

**Affiliations:** 1 Anesthesiology, Hackensack University Medical Center, Hackensack, USA; 2 Anesthesiology, Hackensack Meridian Palisades Medical Center, North Bergen, USA; 3 Internal Medicine, Hackensack Meridian Palisades Medical Center, North Bergen, USA; 4 Pain Management, Hackensack University Medical Center, Hackensack, USA; 5 Pain Management, Riverside Medical Group, Clifton, USA

**Keywords:** neck pain, primary spinal cord tumors, back pain, intramedullary ependymoma, chronic pain management

## Abstract

Intramedullary spinal cord tumors (IMSCT) are a rare subset of neoplasms classified based on anatomical location. The most common presenting symptom is pain; however, the high prevalence of back pain in the general public secondary to common causes including degenerative disc disease or osteoarthritis, makes diagnosing spinal cord tumors a challenge. We present a case of a 43-year-old male with a cervical intramedullary ependymoma to discuss the clinical presentation, diagnosis, and treatment of these spinal tumors.

## Introduction

Primary spinal cord tumors are rare, constituting 2% to 4% of all central nervous system neoplasms, and are classified based on their anatomical locations: extradural, intradural-extramedullary, and intramedullary [1-3]. The least common are Intramedullary spinal cord tumors (IMSCTs), which represent 2% to 5% of spinal tumors and arise from the spinal cord itself [2]. The nonspecific clinical presentation of spinal cord tumors, as well as the prevalence of back pain in the general public, makes diagnosing spinal cord tumors a challenge with possible delays in diagnosis and treatment [3,4]. We present a case of a 43-year-old male with a cervical intramedullary ependymoma to discuss the clinical presentation, diagnosis, and treatment of these spinal tumors.

## Case presentation

A 43-year-old male with a past medical history significant for hyperlipidemia presented complaining of a two-month history of sharp, 10/10 intensity, lower back pain radiating to the anterior thighs and knees bilaterally. The pain worsened with prolonged standing and was associated with subjective weakness of the lower extremities bilaterally. He has experienced no relief with acetaminophen or ibuprofen. He denied numbness or tingling. The patient's current occupation is a porter at an apartment building, responsible for the upkeep of grounds, amenities, and building exteriors. This pain has affected his ability to fully complete his duties at work secondary to pain. Physical examination revealed a full cervical range of motion, limited lumbar range of motion secondary to pain, intact sensation and reflexes of all extremities, non-tender spine, narrow-based gait, and negative straight leg raise. Radiographs of the lumbosacral spine revealed lumbar spondylolysis at the L5 vertebral body, grade 1 isthmic spondylolisthesis at L5-S1, and evidence of degenerative disc disease. The patient was prescribed a course of physical therapy, cyclobenzaprine, and meloxicam.

Despite these interventions, the patient denied interval improvement the following month. He was prescribed a methylprednisolone dose pack for his radicular symptoms and magnetic resonance imaging (MRI) of the spine was ordered. The patient was then referred to a pain management specialist.

At his visit with pain management, the patient reported worsening pain refractory to all medications. The patient had new periscapular and thoracic back pain, significant at night and limiting his sleep. Physical examination was significant for hyperreflexia in quadriceps and triceps surae bilaterally and 1-2 beats of clonus present bilaterally. The patient demonstrated nonantalgic gait and difficulty with a tandem walk. These new symptoms were not consistent with prior physical and imaging, prompting further workup. Upon follow-up, there was continued lower back pain and new left-sided mid-axillary line paresthesias. New physical exam findings included tenderness over the bilateral levator scapulae and upper trapezius. Cervical flexion, lateral bending, and rotation were now limited.

**Figure 1:**
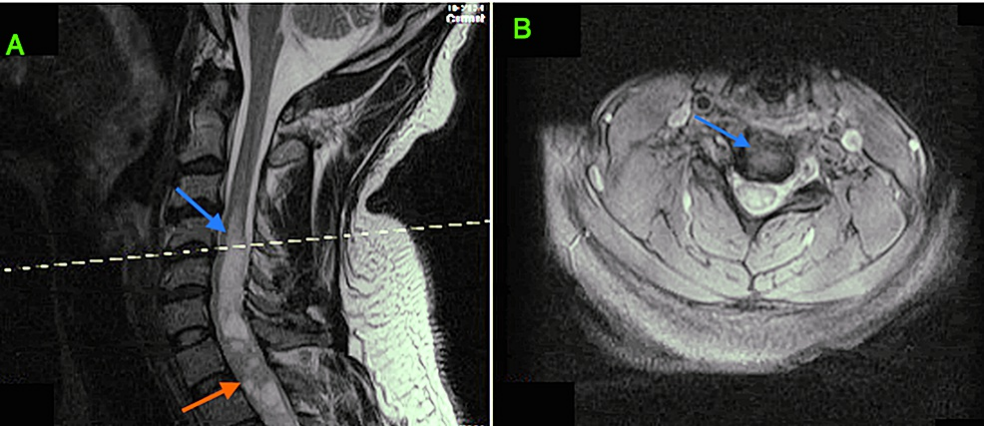
T1-weighted MRI cervical spine. (A) Large syrinx at the C4-C7 (blue arrow). A heterogeneous expanded cord with cystic changes at the C7-T1 (red arrow). (B) Axial cross-sectional cervical spine at C4-C5.

Non-contrast MRI of the spine revealed a large syrinx at the C4-C7 levels and heterogeneous expanded cord with cystic changes at the C7-T1 levels. There was a continuation of the syrinx to the T5 level. MRI with contrast was performed which revealed a 1 cm-wide syrinx at the C4-C6 levels; a 4.3-cm heterogeneously enhancing intramedullary cord lesion from C6 to T1 with internal cystic foci; and a 2.6-cm main enhancing component posterior to C7 and T1 (Figures 1A, 1B).

The patient underwent resection of the intramedullary tumor 16 days after his MRI with posterior arthrodesis from C5 to C6 and instrumentation placement from C5 to T3. Frozen section pathology was consistent with ependymoma. Post-operatively, the patient was started on a dexamethasone taper and transferred to acute inpatient rehabilitation.

Two-month postoperatively, the patient denied any weakness and reported improved calf cramping. He reported having paresthesias in the neck and hyperesthesias in his legs. He had not yet started physical therapy and continued to take gabapentin for neuropathic pain. The patient was pleased with his response to surgical intervention and pain management treatment.

## Discussion

Primary spine tumors are rare neoplasms that can lead to significant morbidity and mortality and are classified as extradural, intradural-extramedullary, or intramedullary [1,5]. The IMSCTs are the rarest of these neoplasms and can lead to severe pain, neurologic deterioration, decreased function, or poor quality of life [4]. Intramedullary tumors originate from the spinal cord, leading to the destruction of gray and white matter [5]. Astrocytomas and ependymomas are the most frequently encountered IMSCTs; astrocytomas are more common in children and ependymomas in adults [4].

The most common presenting symptom for an intramedullary ependymoma is pain, which classically worsens at night and maybe radicular in nature. Differential diagnoses range from benign lesions such as epidermoid cysts or lipomas to tumors such as ependymoma, astrocytoma, ganglioglioma, hemangioblastoma, lymphoma, or metastasis [5].

The tumor may impinge on sensory or motor nerves resulting in paresthesia or weakness, respectively. Sensory symptoms typically precede motor deficits due to the central location of the lesion within the spinal cord. Other signs include ataxia, fasciculations, and decreased deep tendon reflexes. In the later stages of tumor progression, loss of bowel or bladder function may occur [2].

Symptoms of spinal cord tumors may be masked by back pain secondary to common causes including degenerative disc disease or osteoarthritis. In this case, the patient had radicular lumbar back pain with known lumbar spondylolysis that was seen on prior radiographs. However, the following month, the patient presented with new upper motor neuron symptoms including hyperreflexia, nonantalgic gait, and difficulty with tandem walking despite no reports of pain in the neck, mid-back, or upper limbs. These new signs and symptoms warranted additional imaging.

MRI is the preferred modality to image the spine to guide treatment options. Ependymomas typically span multiple vertebral segments, enhance with contrast, are hypo- or isointense on T1-weighted images, and are hyperintense on T2-weighted images [6]. They are found in the central region within the spinal cord, resulting in symmetric expansion, as opposed to astrocytomas that tend to be found more eccentrically [7].

The recommended intervention for intramedullary ependymomas is surgical resection. Resection size and subsequent histological evaluation of the tumor are significant indicators of patient outcomes following surgery [4]. Surgical resection should be performed following a diagnosis of IMSCT, as outcomes correlate with the preoperative neurologic conditions and surgical resection could improve neurologic functions [5]. Intraoperative somatosensory and motor evoked potentials are used to monitor for changes in neurologic function and help guide resection. Ependymomas are distinct tumor and normal spinal cord interface, making gross total resection an option for cure [4,5]. However, given the possibility of recurrence or malignant transformation of spinal ependymomas, it is imperative that the patient be followed regularly with frequent neuroimaging coupled with clinical assessments [5,8].

## Conclusions

Neck and back pain are two of the leading causes of disability, with an annual prevalence of 30% to 80%, respectively, and both cause a significant burden to patients and the healthcare system. While mostly benign, neck or back pain can be an early presenting symptom of more grave illnesses, such as IMSCTs. In such cases, a comprehensive history and physical examination remain the cornerstone in identifying important red flags that may assist toward an early diagnosis. Radiographic evaluation, such as MRI, is necessary to determine the location and extent of tumor involvement and may help to differentiate between lesions. Various types of IMSCT exist and can present with a wide array of signs and symptoms and it is, therefore, essential to recognize symptoms early for prompt diagnosis and treatment.
